# Risk of Bladder Cancer in Patients with Chronic Indwelling Catheters: A Real-World Data Analysis

**DOI:** 10.7150/jca.114223

**Published:** 2025-07-28

**Authors:** Wen-Hsin Tseng, Chang-Hua Lee, Ren-Jie Lin, Chung-Han Ho, Chien-Liang Liu, Steven K. Huang, Allen W. Chiu, Chien-Feng Li, Yow-Ling Shiue

**Affiliations:** 1 Division of Urology, Department of Surgery, Chi Mei Medical Center, Tainan, Taiwan.; 2 Institute of Biomedical Science, College of Medicine, National Sun Yat-Sen University, Kaohsiung, Taiwan.; 3 College of Medicine, Chung Shan Medical University, Taichung, Taiwan.; 4 Department of Medical Research, Chi Mei Medical Center, Tainan, Taiwan.; 5 Department of Urology, Shin Kong Wu Ho-Su Memorial Hospital, Taipei, Taiwan.; 6 Department of Pathology, Chi Mei Medical Center, Tainan, Taiwan.; 7 Institute of Precision Medicine, College of Medicine, National Sun Yat-Sen University, Kaohsiung, Taiwan.

**Keywords:** Bladder Cancer, Chronic Indwelling Catheters, real-word database, Comorbidities, National Health Insurance Research Database

## Abstract

**Introduction:** Bladder cancer is the second most common urological malignancy worldwide, with significant morbidity and mortality. This study investigates the association between chronic indwelling catheter (CIDC) use and bladder cancer risk, particularly in relation to comorbidities and complications.

**Methods:** Taiwan's National Health Insurance Research Database between 2007 and 2018 was used in this study. Patients with CIDC were identified based on records of catheterization on more than six occasions and matched with two patients without CIDC by age, gender, and index date. The outcome, bladder cancer, was identified using ICD-O-3: C67. The incidence rate of bladder cancer was calculated as the number of bladder cancer cases divided by the total follow-up years during the study period. Cox hazards model was also used to adjust with potential confounding variables.

**Results:** A total of 72,971 CIDC patients and 145,942 matched controls were analyzed. The incidence rate of bladder cancer in the CIDC group was 213.29 per 100,000 person-years, significantly higher than 40.4 per 100,000 person-years in the control group with incidence rate ratio: 5.23 (95% CI: 4.60-5.94, p<.0001). After adjusting with confoundings, patients with CIDC show a 5.16-fold higher risk of bladder cancer compared to those without (95% CI, 4.35-6.13, p<.0001). Subgroup analysis revealed a stronger association in younger patients and females. CIDC-related complications, such as urinary tract stones and benign prostatic hyperplasia (BPH), further increased bladder cancer risk.

**Conclusion:** Our findings suggest a strong association between CIDC use and increased risk of bladder cancer, especially among younger patients and those with urological complications such as BPH and urinary tract stones. Additionally, comorbidities including chronic kidney disease, hypertension, and chronic obstructive pulmonary disease may contribute to this elevated risk. Therefore, an integrated healthcare strategy, including monitoring of comorbidities and complications, early cancer detection, and regular risk assessment, is critical for physicians to effectively manage bladder cancer risk in this population.

## Introduction

Bladder cancer ranks as the second most prevalent urological cancer worldwide, with approximately 549,000 new diagnoses and around 200,000 deaths each year 1. For non-muscle-invasive bladder cancer, the recommended treatment involves transurethral resection followed by intravesical chemotherapy. In cases of muscle-invasive bladder cancer, radical cystectomy or radiotherapy combined with systemic chemotherapy is typically advised 2-4. Considering the high recurrence rate of bladder cancer, it can be a thorny and long-lasting challenge for both patients and medical professionals.

Bladder cancer risk factors include tobacco smoking, various occupational and environmental exposures, medications such as cyclophosphamide, and infections like Schistosomiasis 5-7. Among these, chronic bladder inflammation may be a risk factor, potentially triggered by infections (e.g., Schistosomiasis haematobium, recurrent urinary tract infections, Gonorrhea, and other sexually transmitted diseases) or irritants such as urinary stones, indwelling catheters, and benign prostatic hyperplasia (BPH), which may contribute to bladder irritation primarily through urinary retention or recurrent infections, rather than being a direct risk factor itself 8, 9.

In acute-care hospitals, approximately 12-16% of patients will have an indwelling catheter at some point during their admission, while 7.3% of nursing home residents require chronic indwelling catheters (CIDC) 10, 11. Although most catheterizations are temporary, some patients, particularly those with spinal cord injuries, may need long-term catheterization. Previous studies have indicated that patients with CIDC, regardless of spinal cord injury status, face an elevated risk of bladder cancer 12-14, with a particularly high risk associated with catheter use lasting at least 2.9 years 15. However, detailed characterizations of the CIDC patient population have been lacking.

The aim of this study is to evaluate the association between CIDC and bladder cancer risk and to assess whether CIDC is an independent risk factor for bladder cancer by comparing CIDC patients with an age- and gender-matched non-CIDC patients. Additionally, the characteristics of patients who developed bladder cancer between CIDC and non-CIDC groups were analyzed to understand the underlying factors contributing to bladder cancer development in these populations.

## Method and Material

### Data source

The National Health Insurance Research Database (NHIRD) was used in this study. NHIRD includes all claims data from Taiwan's National Health Insurance program since 1997 and about 99% of Taiwan's population is enrolled in this program. The diseases diagnosis in NHIRD was classified using the International Classification of Diseases, Ninth Revision, Clinical Modification (ICD-9-CM) and International Classification of Diseases, Ten Revision, Clinical Modification (ICD-10-CM) codes. The drug prescriptions and the surgical procedures were based on medical expenditure applications. Multiple validation studies have evaluated the accuracy of diagnosis codes in the NHIRD, consistently supporting its reliability for research purposes 16. Moreover, Taiwan cancer registry (TCR) was used to identify the interesting outcome, bladder cancer. TCR is a critical database for evaluating cancer incidence and mortality patterns in Taiwan with high-quality diagnostic and treatment records 17, 18. To encourage research, the Health and Welfare Data Science Center (HWDC) in Taiwan integrates NHIRD and TCR. Under strict governance protocols, HWDC ensures data security and compliance with personal information protection laws.

This study was conducted strictly according to the Declaration of Helsinki. To protect patient confidentiality and prevent ethical violations, all personal identifiers were encrypted before data access. Therefore, individual informed consent was exempted for this study and approved by the Institutional Review Board of Chi Mei Medical Center (IRB No. 11401-015).

### Study subjects

All study subjects were selected from NHIRD between 2007 and 2018, and those were classified as patients with CIDC and those without. The CIDC group was identified based on records indicating CIDC usage on more than six occasions, as documented in their medical expenditure applications. The control group defined as patients without any history of CIDC use throughout the study period. To control the confounding bias, each CIDC patient was matched to two non-CIDC patients based on age, gender, and index date. To investigate the difference of developing new-onset bladder cancer between patients with and without CIDC, all study subjects with bladder cancer before index date have been excluded.

To avoid misclassification bias and minimize treatment-related confounding, individuals with malignancies other than bladder cancer were excluded because their cancer diagnoses could be a confounding factor of CIDC-related bladder cancer risk, such as individuals treated with cyclophosphamide 19. Additionally, patients with diabetes mellitus were excluded due to its potential role as an independent risk factor for bladder cancer and the effects of diabetes medications on cancer risk 20. Other exclusion criteria included patients with autoimmune diseases, such as systemic lupus erythematosus, rheumatoid arthritis, vasculitis, glomerulopathy, or multiple sclerosis, as previous studies indicated these diseases were the risk factor of bladder cancer 9, 21.

### Measurements and outcome

The baseline demographic information, such as age and sex, was analyzed in this study. Age was categorized into three groups of <50, 50~64, and ≧65 years. After matching for age and sex, comorbidities and complications were considered primary adjustment factors to assess the risk of developing bladder cancer between patients with CIDC and those without.

All comorbidities and complications were identified using ICD-9-CM or ICD-10-CM codes. Comorbidities, based on 1-year inpatient and outpatient records before the index date, included hypertension, hyperlipidemia, chronic kidney disease (CKD), chronic obstructive pulmonary disease (COPD), and liver disease. Complications were defined as conditions occurring within one year before bladder cancer diagnosis or the last observation date, including benign prostatic hyperplasia (BPH), urinary tract infections (UTIs), and urinary tract stones (renal and bladder stones). All diagnostic codes were provided in the Supplementary Table.

The major outcome of this study was bladder cancer, identified using ICD-O-3: C67 from the Taiwan Cancer Registry. All study subjects were right censored on December 31, 2018 or at the time of bladder cancer diagnosis, death, withdrawal from the insurance program, or loss to follow-up. The maximal follow-up period were 12 years.

### Statistical analysis

Categorical variables, including of age, sex, comorbidities, complication, and bladder cancer, were presented as frequency with percentage, and the difference between patients with CIDC and those without was analyzed using Pearson's Chi-square test. Mean differences of duration from index date to bladder cancer incidence were evaluated using Student's t-test. The incidence rate of bladder cancer for each group was expressed as rates per 100,000 person-years and calculated as the number of bladder cancer cases divided by the total follow-up years during the study period. The incidence rate ratio (IRR) of bladder cancer was estimated using Poisson regression with total person-time as an offset variable. Additionally, Kaplan-Meier curves were plotted to illustrate the cumulative incidence of bladder cancer in each group, with trends compared using the log-rank test. Selected risk factors were analyzed using Cox proportional hazards models with covariates incorporated to adjust with potential confounding variables. The subgroup analysis, illustrated using forest plot, also presented to estimate the risk ratio of bladder cancer between patients with and without CIDC among different sex and age group. Statistical significance was set as a P-value of <0.05. All analyses were conducted using the Statistical Analysis System (SAS) software (version 9.4; SAS Institute, Inc., Cary, NC, USA).

## Results

Table 1 presented baseline information between patients with and without CIDC. Of all 218,913 study subjects, 72,971 patients with CIDC and 145,942 patients without CIDC were analyzed in this study. Approximately 60.61% of these patients were male and 71.96% of those were over 65 years old. After matching with age and sex, those patients with CIDC had statistically significant higher percentage in hypertension (36.15% vs. 35.66%, p=0.0256) and COPD (10.42% vs. 9.15%, p<.0001). Hyperlipidemia show a lower percentage in patients with CIDC compared to the patients without CIDC (6.71% vs. 9.23%, p<0.0001). Additionally, patients with CIDC presented significantly higher complication than patients without CIDC in BPH (26.45% vs. 12.01%, p<.0001), UTI (57.95% vs. 8.92%, p<.0001), and urinary tract stones (4.31% vs. 0.95%, p<.0001). During the follow-up period, 1129 patients were newly diagnosed with bladder cancer, including 1.10% of patients in the CIDC group and 0.22% in the control group. For time to develop bladder cancer, patients with CIDC presented shorter time than patients without CIDC (2.63±2.80 years vs. 3.95±2.87 years, p<.0001).

The incidence rates of bladder cancer were 213.29 per 100,000 person-years in the CIDC group, which was significantly 5.23-folds (95% C.I.: 4.60-5.94, p<.0001) higher than patients without CIDC (40.84 per 100,000 person-years). Patients with CIDC had incidence rate of bladder cancer for 273.37 and 113.84 per 100,000 person-years in males and females, respectively. Patients who aged 65 years and older had higher bladder cancer incidence rates of 259.83 and 62.73 per 100,000 person-years in the CIDC and non-CIDC groups, respectively. However, among different age groups, patients under 50 years old presented the highest IRR at 17.97(6.35-50.80), with p<0.0001. Moreover, patients with selected comorbidities and complications in the CIDC group exhibited a higher incidence of bladder cancer compared to those without CIDC. Especially, CKD patients show approximately a ten-fold IRR of bladder cancer between patients with CIDC and those without (IRR: 10.36, 95% C.I.: 1.25-86.04, p=0.0304). COPD Patients with CIDC also show the incidence rate of bladder cancer for 232.74 per 100,000 person-years (Table 2).

**Figure 2:**
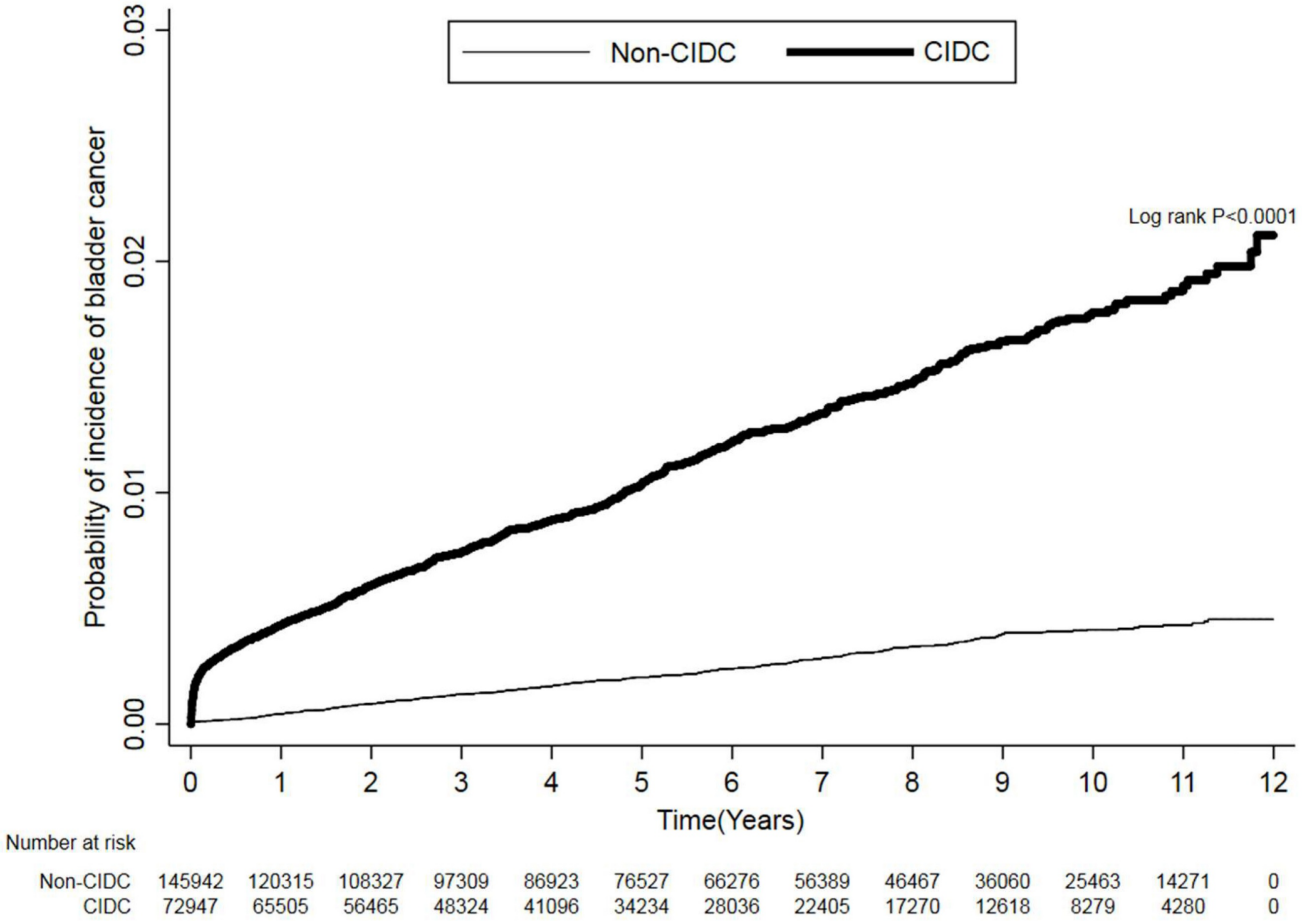
Cumulative Incidence of Bladder Cancer in Patients With and Without CIDC using Kaplan-Meier approach.

Kaplan-Meier plots (Figure 2) demonstrate that patients in the CIDC group had a higher trend of developing bladder cancer than non-CIDC group during the study periods. The log-rank test was p<.0001. Table 3 presents the hazard ratio of bladder cancer between patients with and without CIDC after adjusting with the comorbidities and complications. Patients with CIDC show a significant higher risk of bladder cancer compared with patients without CIDC (hazard ratio (HR): 5.16, 95% CI, 4.35-6.13, p<0.0001). Additionally, study subjects with complications of BPH and urinary tract stones had significant higher risk of developing bladder cancer compared to those without these complications (HR: 1.92, 95% CI: 1.50-2.45, p<.0001 for BPH; HR: 2.58, 95% CI: 1.52-4.37, p=0.0004 for urinary tract stones).

**Figure 3:**
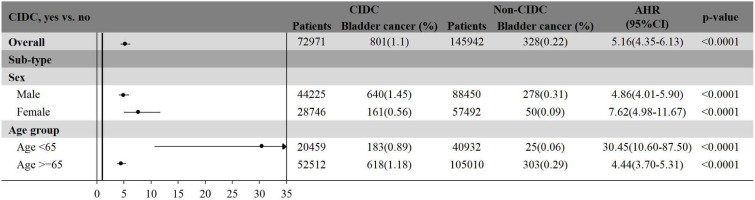
The subgroup analysis of bladder cancer risk among patients with and without CIDC, stratified by sex and age group

The subgroup analysis illustrated the risk of bladder cancer among patients with and without CIDC, stratified by sex and age group (Figure 3). In males, patients with CIDC had 4.86-fold higher risk of developing bladder cancer compared to those without CIDC (95% CI: 4.01-5.90, p<.0001). Similarly, female patients with CIDC exhibited a significantly increased risk of bladder cancer with HR of 7.62 (95% CI: 4.98-11.67, p<.0001) compared to those without CIDC. When stratified by age, patients who are younger than 65-years show the highest relative risk of bladder cancer between CIDC and non-CIDC groups (HR: 30.45, 95% CI: 10.60-87.50, p<.0001). In contrast to patients aged 65 years and older, those with CIDC had 4.44-fold increased risk of bladder cancer compared to those without CIDC (95% CI: 3.70-5.31, p<.0001).

## Discussion

In this large population-based cohort study, we found a significant association between CIDC and an increased risk of bladder cancer. Patients with CIDC had a more than 5-fold higher risk of developing bladder cancer compared to non-CIDC patients. The incidence rate of bladder cancer in the CIDC group was higher than in the non-CIDC group, especially among patients who aged younger than 65 years. Subgroup analysis further indicated that females with CIDC show about 7-fold increased risk of bladder cancer compared to those without CIDC. Additionally, complications, such as BPH and urinary tract stones, were associated with an increased risk of bladder cancer in CIDC patients. These findings emphasize the importance of regular monitoring and early detection of bladder cancer in CIDC patients, particularly those with underlying urological conditions.

In our study, more patients had comorbidities of hypertension and COPD in CIDC group. Urinary retention which is the main indication of urinary catheter is associated with several chronic conditions. A system review indicated that in patients with COPD, inhaled anticholinergics can increase the risk of acute urinary retention, particularly in older patients with BPH 22-24. Hypertension might increase the catheter requirement, as it is likely to impact the nervous system (like stroke or spinal cord injury) and cause neurogenic bladder, which requires long-term catheter use 25. Moreover, Eicher et al. reported that certain antihypertensive medication like Nicardipine, a calcium channel blocker, has been linked to urination disorders in some patients 26.

There have been some researches suggesting a complex relationship between hypertension and bladder cancer. A large-scale cohort study of 79,236 people found a positive association between hypertension and subsequent bladder cancer development, with a 32% increased risk overall and a more pronounced effect in women 27. The other meta-analysis identified bladder cancer as one of several organ-specific cancers associated with hypertension, suggesting that hypertension-related angiogenesis factors may play a role in cancer initiation, though bladder cancer had a weaker positive association (RR: 1.21; CI: 0.97-1.50; I2: 0%) 28. On the other hand, COPD and bladder cancer share the same risk factor of cigarette use, which may explain why there are more people with COPD developed bladder cancer. Naka et al. has studied 217 people who were newly diagnosed urothelial cancer patients and found the prevalence is high, with 38.6% displaying FEV1/FVC < 70% 29.

In our study, among the 801 patients who developed bladder cancer in CIDC group, UTIs comprised of 476 people, which is significantly more than the population controls. One large retrospective study in Canada has indicated that cancer-specific mortality was significantly association with chronic catheterization and the number of symptomatic UTIs. The risk of bladder cancer-specific mortality was more than 8-fold higher in patients with CIDC than in the general population 14. The possible mechanism by which UTIs lead to bladder cancer has been widely researched. X. Huang et al. revealed that the inflammatory microenvironment and the urinary microbiome might play a role in the initiation and progression of bladder cancer. The study indicated that bladders harbour distinct commensal microorganisms. In healthy bladders (eubiosis), the metabolic byproducts of commensal microorganisms can prompt the host to release anti-inflammatory cytokines, which help sustain balance. Furthermore, NOD-like receptors (NLRs) defend against microbial invasion. However, under pathological conditions (dysbiosis), harmful microbial populations outgrow the native microbiota, triggering inflammation. Persistent inflammation harms epithelial cells by generating reactive oxygen species (ROS) and reactive nitrogen species (RNS), resulting in cell death and further weakening of the epithelial barrier. Prolonged cycles of inflammation can eventually lead to cancer development 30.

The major strength of this study was our results derived from a nationwide real-world database with detailed longitudinal medical information. Therefore, the incidence risk of bladder cancer among patients with CIDC could be precisely estimated across various comorbidities and complications based on large sample sizes. However, several limitations should be mentioned in this study. First, NHIRD did not include the information on some critical risk factors of bladder cancer, such as occupational exposures, laboratory data, and lifestyle factors (smoking and alcohol consumption). Moreover, the database does not include information on physical disabilities, such as spinal cord injury. Patients with spinal cord injury may have an elevated risk of bladder cancer independent of catheter use 12, 13. If such conditions were disproportionately presented in the CIDC group, they could be unmeasured confounders and partially explain the observed association. Additionally, the potential misclassification bias may exist according to the variations in the definition of comorbidities using the ICD diagnostic codes. However, multiple validation studies have supported the accuracy of NHIRD data in previous research 31. Another limitation is that the effect of the duration of CIDC on bladder cancer could not be assessed due to the lack of the exact records on the initiation of chronic catheterization among the CIDC patients. In addition, the lower prevalence of hyperlipidemia in CIDC patients may be due to underdiagnosis or underreporting in patients with severe underlying conditions or disabilities who require long-term catheterization, where management of chronic metabolic conditions like hyperlipidemia may be lower clinical priority. Similar patterns have been observed in multimorbid and functionally impaired populations, where chronic metabolic conditions such as hyperlipidemia may be underdiagnosed due to competing care priorities 32, 33. Finally, details about catheter type, material, and management practices were unavailable, which may affect the risk of bladder cancer development. To improve risk assessment of bladder cancer, future research should consider to evaluate the long-term effects of CIDC duration, catheter type, and infection burden on bladder cancer incidence using prospective study design.

## Conclusions

In conclusion, our study demonstrated that CIDC is a significant risk factor for bladder cancer, especially among younger patients and those with urological complications such as BPH and urinary tract stones. Additionally, comorbidities including CKD, hypertension, and COPD may increase the bladder cancer development in CIDC patients. Therefore, an integrated healthcare strategy, including monitoring of comorbidities and complications, early cancer detection, and regular risk assessment, is critical for physicians to effectively manage bladder cancer risk in this population.

## Supplementary Material

Supplementary table.

## Figures and Tables

**Figure 1:**
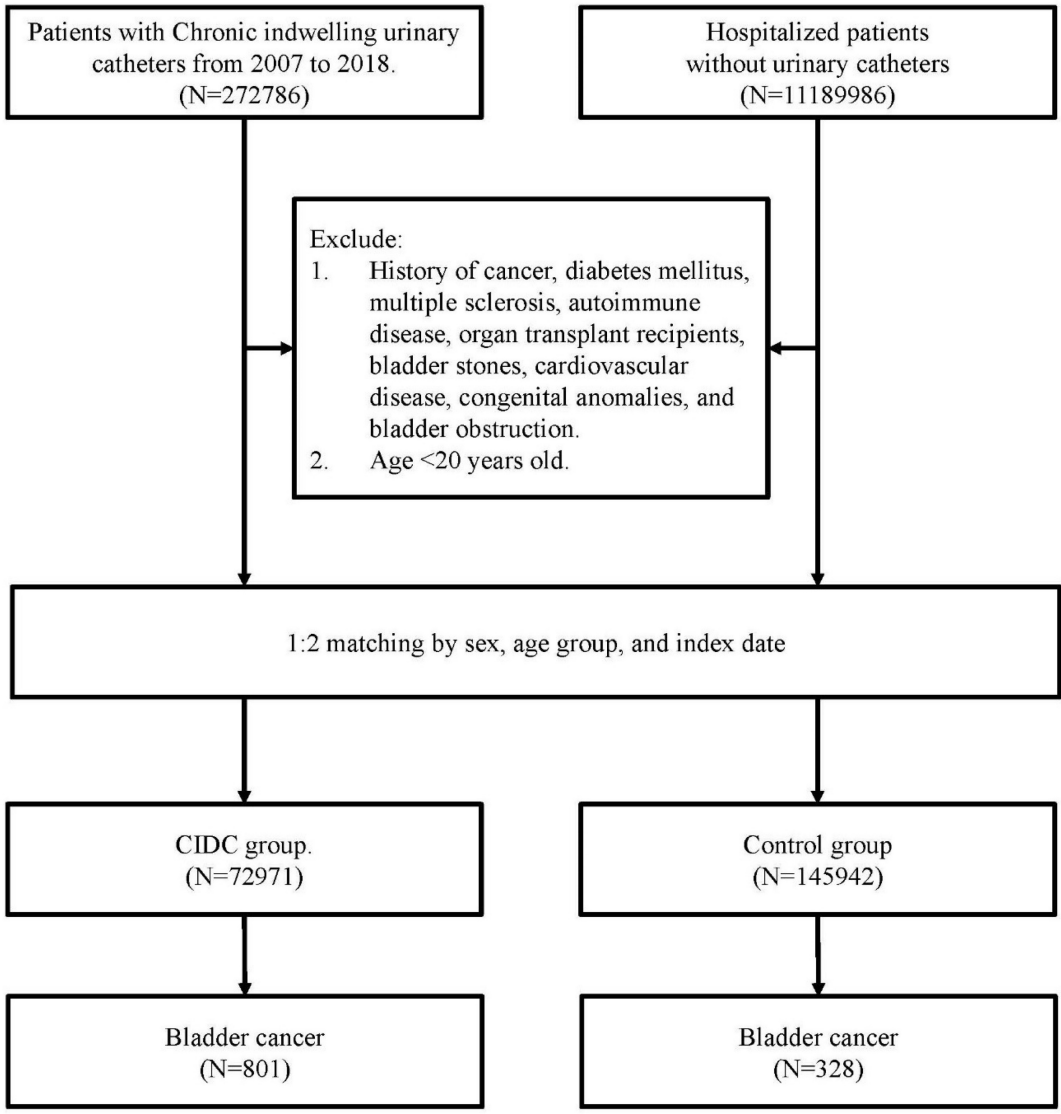
The flowchart of study subjects' selection.

**Table 1:** The characteristics of patients with and without CIDC.

	Overall (N=218913)	CIDC (N=72971)	Control (N=145942)	p-value
Gender, n (%)				
Male	132675(60.61)	44255(60.61)	88450(60.61)	1.0000
Female	86238(39.39)	28746(39.39)	57492(39.39)	
Age group, n (%)				
<50	25066(11.45)	8369(11.47)	16697(11.44)	0.9628
50-65	36325(16.59)	12090(16.57)	24235(16.61)	
≧65	157522(71.96)	52512(71.96)	105010(71.95)	
Comorbidity, n (%)				
Hypertension				
Yes	78426(35.83)	26378(36.15)	52048(35.66)	**0.0256**
No	140487(64.17)	46593(63.85)	93894(64.34)	
Hyperlipidemia				
Yes	18370(8.39)	4895(6.71)	13475(9.23)	**<0.0001**
No	200543(91.61)	68076(93.29)	132467(90.77)	
CKD				
Yes	1253(0.57)	422(0.58)	831(0.57)	0.7945
No	217660(99.43)	72549(99.42)	145111(99.43)	
COPD				
Yes	20961(9.58)	7606(10.42)	13355(9.15)	**<0.0001**
No	197952(90.42)	65365(89.58)	132587(90.85)	
Liver disease				
Yes	11248(5.14)	3790(5.19)	7458(5.11)	**0.4036**
No	207665(94.86)	69181(94.81)	138484(94.89)	
Complication, n (%)				
BHP				**<0.0001**
Yes	36821(16.82)	19300(26.45)	17521(12.01)	
No	182092(83.18)	53671(73.55)	128421(87.99)	
UTI				**<0.0001**
Yes	55311(25.27)	42286(57.95)	13025(8.92)	
No	163602(74.73)	30685(42.05)	132917(91.08)	
Urinary Tract Stones				**<0.0001**
Yes	4530(2.07)	3143(4.31)	1387(0.95)	
No	214383(97.93)	69828(95.69)	144555(99.05)	
Bladder cancer, n (%)	1129(0.52)	801(1.10)	328(0.22)	**<0.0001**
Time to event (year), mean±SD	3.01±2.88	2.63±2.80	3.95±2.87	**<0.0001**
Death, n (%)	93429(42.68)	41892(57.41)	51537(35.31)	**<0.0001**

*Patients with CIDC and those without were 1:2 matched by age, sex, and index date. CIDC: chronic indwelling catheter; CKD: chronic kidney disease; COPD: chronic obstructive pulmonary disease; BPH: benign prostatic hyperplasia; UTI: urinary tract infections; SD: standard deviation.

**Table 2:** The incidence rate of bladder cancer in CIDC patients and non-CIDC patients.

	CIDC				Non-CIDC				IRR(95% CI)	p-value
	Patients	Bladder cancer (%)	PY	Incidence rate^*^	Patients	Bladder cancer (%)	PY	Incidence rate^*^		
Overall	72971	801(1.10)	375540.55	213.29	145942	328(0.22)	803207.90	40.84	5.23(4.60-5.94)	**<0.0001**
Gender										
Male	44225	640(1.45)	234118.86	273.37	88450	278(0.31)	487488.55	57.03	4.80(4.17-5.52)	**<0.0001**
Female	28746	161(0.56)	141421.69	113.84	57492	50(0.09)	315719.35	15.84	7.19(5.24-9.88)	**<0.0001**
Age group										
<50	8369	32(0.38)	61969.52	51.64	16697	4(0.02)	139155.08	2.87	17.97(6.35-50.80)	**<0.0001**
50-65	12090	151(1.25)	75722.72	199.41	24235	21(0.09)	181062.92	11.6	17.20(10.89-27.14)	**<0.0001**
>=65	52512	618(1.18)	237848.30	259.83	105010	303(0.29)	482989.91	62.73	4.15(3.61-4.76)	**<0.0001**
Comorbidity										
Hypertension	26378	315(1.19)	121675.22	258.89	52048	147(0.28)	245074.39	59.98	4.32(3.55-5.25)	**<0.0001**
Hyperlipidemia	4895	67(1.37)	24370.14	274.93	13475	48(0.36)	72999.29	65.75	4.18(2.89-6.06)	**<0.0001**
CKD	422	6(1.42)	501.25	1197.00	831	1(0.12)	860.82	116.17	10.36(1.25-86.04)	**0.0304**
COPD	7606	73(0.96)	31365.91	232.74	13355	38(0.28)	42857.49	88.67	2.63(1.78-3.89)	**<0.0001**
Liver disease	3790	34(0.90)	19459.82	174.72	7458	15(0.20)	37294.06	40.22	4.35(2.37-7.98)	**<0.0001**
Complication										
BHP	19300	381(1.97)	96377.73	395.32	17521	141(0.8)	102242.43	137.91	2.87(2.36-3.48)	**<0.0001**
UTI	42286	329(0.78)	187867.65	175.12	13025	74(0.57)	55421.74	133.52	1.31(1.02-1.69)	**0.0350**
Urinary Tract Stones	3143	67(2.13)	16825.59	398.2	1387	21(1.51)	8315.09	252.55	1.58(0.97-2.57)	0.0687

* per 100000 person-years; * CIDC: chronic indwelling catheter; CKD: chronic kidney disease; COPD: chronic obstructive pulmonary disease; BPH: benign prostatic hyperplasia; UTI: urinary tract infections; PY: person-years; IRR: incidence rate ratio; CI: confidence interval. Note: Patients may have more than one comorbidity or complication; thus, the total number across conditions may exceed the total number of individuals in each group.

**Table 3:** The risk of bladder cancer in CIDC patients and non-CIDC patients.

	Crude HR (95% C.I.)	p-value	Adjusted HR (95% C.I.)	p-value
CIDC, yes vs. no	5.73(4.93-6.66)	**<0.0001**	5.16(4.35-6.13)	**<0.0001**
Comorbidity				
Hypertension	1.14(0.96-1.34)	0.1315	1.21(0.97-1.51)	0.0982
Hyperlipidemia	0.90(0.70-1.17)	0.4360	1.08(0.77-1.51)	0.6646
CKD	3.49(1.02-11.93)	**0.0459**	2.53(0.50-12.97)	0.2646
COPD	0.86(0.65-1.14)	0.2920	0.73(0.51-1.04)	0.0800
Liver disease	0.90(0.62-1.29)	0.5591	1.07(0.67-1.7)	0.7806
Complication				
BHP	3.21(2.64-3.91)	**<0.0001**	1.92(1.50-2.45)	**<0.0001**
UTI	3.25(2.68-3.94)	**<0.0001**	1.02(0.79-1.32)	0.8840
Urinary Tract Stones	4.60(3.02-7.00)	**<0.0001**	2.58(1.52-4.37)	**0.0004**

* CIDC: chronic indwelling catheter; CKD: chronic kidney disease; COPD: chronic obstructive pulmonary disease; BPH: benign prostatic hyperplasia; UTI: urinary tract infections; HR: hazard ratio; CI: confidence interval.

## Abbreviations

CIDC: Chronic Indwelling Catheter
BPH: Benign Prostatic Hyperplasia
NHIRD: National Health Insurance Research Database
ICD-9-CM: International Classification of Diseases, Ninth Revision, Clinical Modification
ICD-10-CM: International Classification of Diseases, Ten Revision, Clinical Modification
TCR: Taiwan cancer registry
HWDC: Health and Welfare Data Science Center
CKD: Chronic Kidney Disease
COPD: Chronic Obstructive Pulmonary Disease
BPH: Benign Prostatic Hyperplasia
UTIs: Urinary Tract Infections
IRR: Incidence Rate Ratio
SAS: Statistical Analysis System
NLRs: NOD-like receptors
ROS: Reactive Oxygen Species
RNS: Reactive Nitrogen Species
