# Supplementary material for: Risk of Bladder Cancer in Patients with Chronic Indwelling Catheters: A Real-World Data Analysis
Source: J Cancer. 2025 Jul 28;16(11):3464–72. doi: 10.7150/jca.114223 (PMC12374942; doi:10.7150/jca.114223)
Supplement: Supplementary file 1 — Supplementary table. [file jcav16p3464s1.pdf]

Supplementary Table. All diagnostic codes in this study

	ICD-O-3	ICD-9-CM	ICD-10-CM
**Bladder cancer**	C67		
**Exclude**			
Cancer		140-208	C00-C97
DM		250	E08-E13
Multiple sclerosis		340	G35
Autoimmune disease			
Systemic lupus erythematosus		710.0	M32
Rheumatoid arthritis		714	M05.1-M05.0, M05.6-M05.8, M06.0, M06.2, M06.3, N06.8, M06.9, M08.0, M08.2-M08.4, M12.0
Vasculitis		446.4	M31.3
Glomerulopathy		580-589	N00-N08
Organ transplant recipients		E878.0	Y83.0
Bladder stones		594.0, 594.1	N21.0
CVD		430-438, 362.34	G45, G46, I60-I69, H34.0
Congenital anomalies		753.6, 753.8	Q64
Bladder obstruction history		596.0	N32.0
**Comorbidity**			
Hypertension		401-405	I10-I15
Hyperlipidemia		272	E78

CKD	585	N18
COPD	491, 492, 496	J41-J44
Liver disease	070, 570-573, 456.0, 456.1, 456.2, V42.7	K70-K76, B18, Z94.4, I85.0, I85.9, I86.4, I98.2
**Complication**		
BPH	600	N40
UTI	599.0	N39.0
Renal and bladder stone	592.0, 594.0, 594.1	N20.0, N21.0

*DM: diabetes mellitus; CVD: cardiac vascular disease; CKD: chronic kidney disease; COPD: chronic obstructive pulmonary disease; BPH: benign prostatic hyperplasia; UTI: urinary tract infection.
